# The stability of multidimensional subclinical apathy during a pandemic and its relations to psycho-behavioral factors

**DOI:** 10.1038/s41598-022-06777-5

**Published:** 2022-02-21

**Authors:** Giulia Lafond-Brina, Anne Bonnefond

**Affiliations:** 1 INSERM U1114, Pôle de Psychiatrie, 1 Place de l’Hôpital, 67000 Strasbourg, France; 2 University of Strasbourg, Strasbourg, France

**Keywords:** Psychology, Signs and symptoms

## Abstract

Apathy is a clinical symptom prevalent in many neuropsychiatric pathologies. Subclinical apathy is found in 35% of the general population. Despite high prevalence and negative consequences, underlying mechanisms are poorly understood, perhaps because the concept of apathy is one-dimensional. The current investigation aims to address the incidence of multidimensional apathetic trait in three distinct forms in a student population, to specify its determinants and to evaluate its stability during a global pandemic. Two online surveys, conducted 1 year apart on two separate cohorts of university students, with qualitative measures and validated scales. The final analysis included, respectively, 2789 and 1678 students. The three forms of apathetic trait were present, with the same debilitating consequences as apathetic symptom but independent determinants. Executive apathy was predicted by depressive symptoms, emotional apathy by motivational deficit and initiative apathy comprised a mixed executive-emotional form and a pure deficit of action initiation. The three forms of subclinical apathy remained similar in the context of increased depressive symptoms due to a global pandemic. This study confirmed the presence and independence of three forms of subclinical apathy in healthy students, which remained similar even in the light of increased depressive scores. These results shed light on cognitive and neuronal mechanisms underlying multidimensional apathy, allowing new, targeted treatments.

## Introduction

Did you ever hear a teacher call a lazy student ‘apathetic’? Or a psychologist call a patient ‘apathetic’ when they seem indifferent? In everyday language, it can be used in many contexts, but the term ‘apathy’ also refers to a clinical symptom^1^. Specifically, apathy is defined by quantitative decrease in goal-directed activity in comparison to the person’s previous level of functioning (International consensus group—Robert et al.^2^). Different terms, depending mainly on the pathology, are used (‘avolition’, ‘aboulia’, ‘apragmatism’…) but all describe the same clinical symptom^3,4^. Apathy is a transnosographic symptom, prevalent in many neurological and psychiatric pathologies, and almost half of patients suffer from it^5^. It is an important source of burden, affecting both personal and occupational life^6–8^.

**Figure 1:**
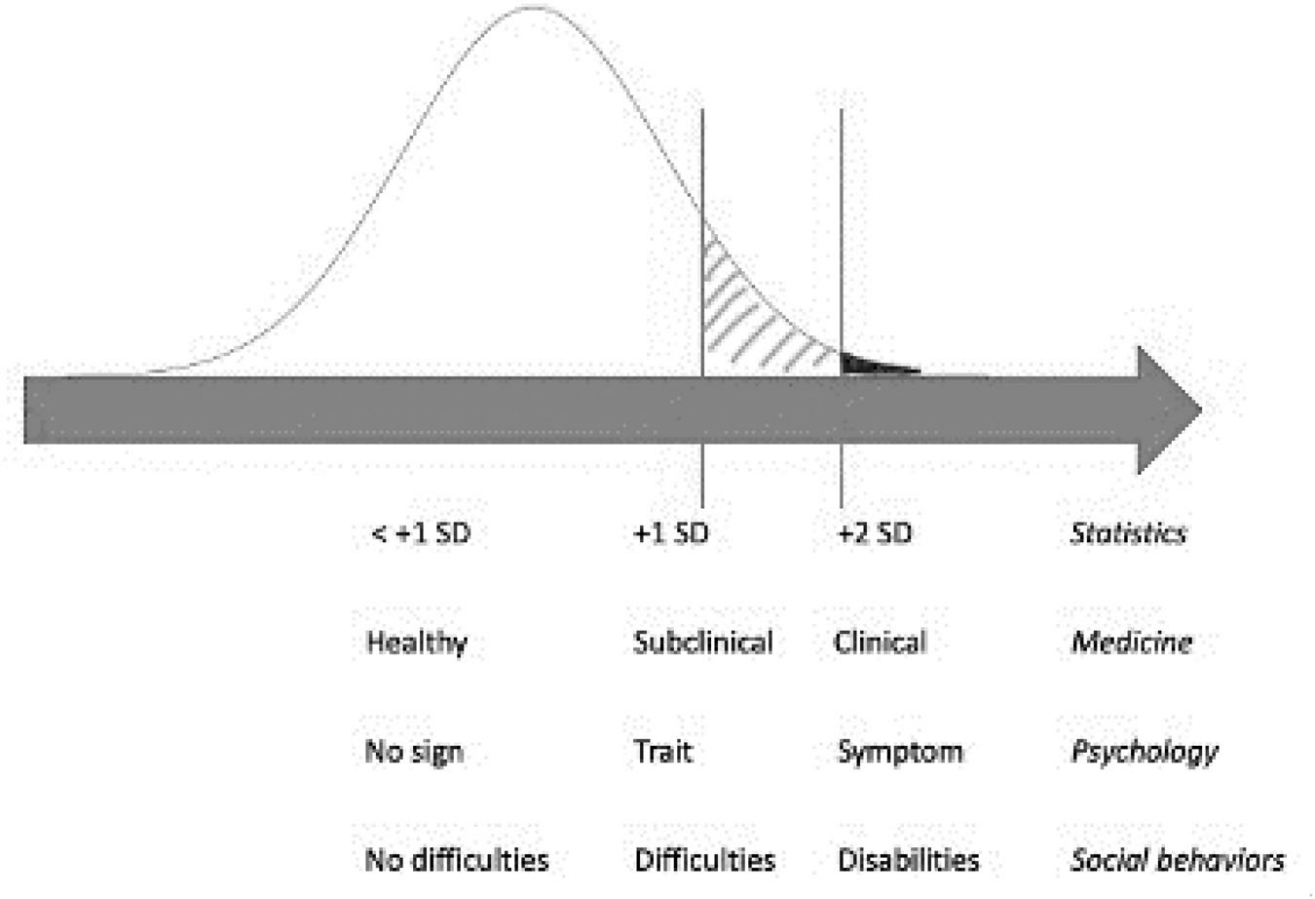
Differences of subclinical and clinical concepts in four fields: statistics, medicine, psychology, and social behaviors.

In the general population, about 2% of healthy young people^9^ and about 6% of healthy older people^10^ suffer from apathy, and show apathy scores similar to those of patients (i.e., more than two standard deviations from the population mean) and the same disability. In a recent article from Ang et al.^11^, about 35% of a sample of healthy adults presented a milder apathy, often described as subclinical apathy (more than one standard deviation from the mean)^11^. This may represent a trait: i.e., a middle position between asymptomatic healthy people and patients with disabling symptoms. Figure 1 illustrates the differences of subclinical and clinical concepts. Interestingly, it has been shown that subclinical apathy negatively impacts the same personal and occupational dimensions as the clinical symptom: the stronger the apathetic trait, the greater the depression, fatigue and isolation, and the lower the hedonia, empathy and academic success^12,13^.

However, despite its high prevalence and negative consequences, no pharmacological or non-pharmacological treatments exist^14^, and the underlying mechanisms of apathy are poorly understood^2,15^. One reason for this may be an overlap in diagnostic criteria between apathy and depressive symptoms, which remains a source of confusion. Yet there is evidence that apathy and depressive symptoms are distinct. Clinically, people can suffer from apathy without being depressed or present depressive symptoms without apathy^4^. Neuroimaging results point in the same direction, revealing the involvement of distinct cerebral networks in apathy and depressive symptoms^16,17^. A further explanation may be that a one-dimensional concept of apathy still prevails^18,19^, despite clinical observations and fMRI findings in brain-damaged patients that both support the idea that apathy is multidimensional^20–22^.

In light of these results, Levy and Dubois^23^ proposed a multidimensional model of apathy, distinguishing three forms: cognitive/executive, emotional and auto-activation/initiative. According to this model, executive disorders underlie the cognitive form of apathy. Patients with executive apathy report difficulty in planning new action, switching between tasks and focusing on an activity. It may be related to lesions of the dorsolateral prefrontal cortex and the cognitive territory of the basal ganglia. Emotional apathy, characterized by difficulty in expressing and experiencing emotion, empathy, and interest, could be due to motivational disorder. Dysfunctions or lesions in the orbital and medial prefrontal cortex and limbic territories of the basal ganglia may underlie this. Finally, the initiative form, associated with more severe symptoms, is often described as mental emptiness with difficulty in thinking of new things, being spontaneous and initiating social contact, and may be a mixed form, with both motivational and executive difficulties. Lesions or dysfunctions may affect both the cognitive and limbic territories of the basal ganglia or the anterior cingulate cortex.

Although this multidimensional concept was confirmed in patients suffering from neurodegenerative disease^15,24,25^ and schizophrenia^26^, no studies addressed the three distinct forms of apathetic trait in a healthy population. Confirming its presence in healthy people (i.e., without other associated pathology) and identifying specific determinants for each form would help specify and investigate the respective neural mechanisms.

From this perspective, the present investigation was divided into two studies. The first study had three main aims: (1) to assess the presence of the three forms of apathy (executive, emotional and initiative) in a young and healthy population; (2) to assess links between each form and various factors relating to sociodemographic characteristics, past and ongoing education, general functioning, and psychopathology and (3) to determine which of these factors best predicts each form of apathy. Following the conclusions given by the first study and the emergence of COVID-19 pandemic, we conducted a second study, with three main purposes: (1) to confirm the stability across a year of the distribution of multidimensional subclinical apathy tested in a different sample of young students; (2) to replicate two major predictors (depression and gender) highlighted in Study 1 and (3) to evaluate the contextual stability of subclinical apathy and how it differs from depression in a student population, considering the pandemic context, which is known to be associated with increased depression in young populations^27–29^.

## Results

The present investigation was based on two studies conducted 1-year apart. Its objectives were to address the incidence of apathetic trait in three distinct forms (executive, emotional and initiative) in a young and healthy student population and to evaluate its stability over time and during a global pandemic. The first study also determined the correlates and predictors of each form from several factors relating to sociodemographic characteristics, past and present education, general functioning, and psychopathology.

### Study 1

#### Presence of the three forms of apathy

Mean executive apathy score was 11.43 ± 4.77 (range: 0–24). Mean emotional apathy score was 7.46 ± 4.24 (range: 0–24). Mean initiative apathy score was 10.20 ± 3.94 (range: 0–24). The other results on the validated questionnaires are summarized in Table 1.

**Table 1:** Descriptive results of the validated questionnaires.

Questionnaire	Mean ± standard deviation	Minimum–maximum
DAS total	29.07 ± 8.70	6–64
BDI-II	8.31 ± 6.45	0–38
RSE	27.5 ± 6.21	10–40
TEPS anticipatory	44.8 ± 7.30	10–60
TEPS consummatory	37.2 ± 6.22	8–48

*DAS* The Dimensional Apathy Scale (Radakovic and Abrahams^47^), *BDI-II* The Beck Depression Inventory II (Beck et al. ^52^), *RSE* The Rosenberg Self-Esteem Scale (Rosenberg ^53^), *TEPS* The Temporal Experience of Pleasure Scale (Gard et al. 2006).

Among the respondents, 21.2% had an executive apathy trait (cut-off: 16), 16.2% an emotional apathy trait (cut-off: 12) and 20.9% an initiative apathy trait (cut-off: 14); 3.2% had an executive apathy symptom (cut-off: 21), 4.8% an emotional apathy symptom (cut-off: 16) and 3.6% an initiative apathy symptom (cut-off: 18) (see Supplementary data 1 online).

**Figure 2:**
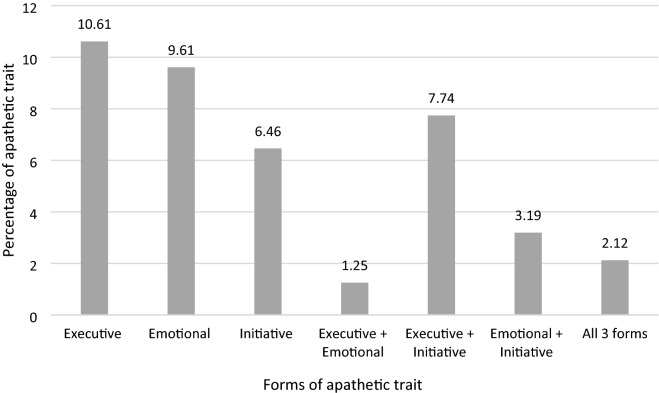
Combinations of multidimensional apathetic trait in the sample.

Specifically, 28.68% of respondents presented a single form of apathetic trait, 11.68% combined two forms, and 2.12% had all three forms. Figure 2 illustrates the percentage of combinations for the three forms of apathetic trait.

Among the respondents, 9.44% presented a single form of apathetic symptom (2.55% executive, 4.20% emotional and 2.69% initiative apathy), 1.08% had mixed symptoms (0.18% executive and emotional forms, 0.43% executive and initiative forms, and 0.47% emotional and initiative forms), and none (0.00%) presented all three symptoms together.

Analyses of correlations between the three forms of apathetic trait showed that executive and emotional apathy were no correlated (r = − 0.065; p > 0.05), whereas initiative apathy was positively correlated with both executive (r = 0.405; p < 0.001) and emotional apathy (r = 0.205; p < 0.001).

#### Identification of variables correlated with each form of apathy

##### Executive apathy

*Sociodemographic* ANOVAs on executive apathy scores revealed that no sociodemographic factor showed a significant, practical effect.

*Education* ANOVAs on executive apathy scores revealed significant, small effects of type of field (F(4,2784) = 20.8; p < 0.001; η^2^ = 0.029), level of study (F(5,2783) = 9.81; p < 0.001, η^2^ = 0.017), Bachelor’s degree with honours (F(5,2783) = 7.26; p < 0.001; η^2^ = 0.013) and choice of field (F(3,2785) = 26.6; p < 0.001; η^2^ = 0.028). More precisely, Bonferroni tests showed that executive apathy scores were higher for students in the arts and literature field than for those in the other fields (t(2784) = 8.687; p < 0.001; d = 0.16), for first- and second-year undergraduates than PhD degree students (t(2783) = 5.94; p < 0.001; d = 0.11), for students without honours than for those with honours in their Bachelor’s degree (t(2783) = 4.51; p < 0.001; d = 0.09) and for students in a field they had chosen but did not like, compared to those who had chosen their field and liked it (t(2785) = 8.77; p < 0.001; d = 0.17).

*General functioning* ANOVAs on executive apathy scores revealed significant, large effects of daily pleasure (F(3,2785) = 148; p < 0.001; η^2^ = 0.137) and dynamism (F(1,2787) = 324; p < 0.001; η^2^ = 0.104); moderate effects of isolation (F(2,2786) = 82.1; p < 0.001; η^2^ = 0.056) and small effects of number of initiated social contacts (F(3,2785) = 23.6; p < 0.001; η^2^ = 0.025), independence (F(1,2787) = 51.7; p < 0.001; η^2^ = 0.018) and leisure (F(1,2787) = 47.5; p < 0.001; η^2^ = 0.017). Indeed, executive apathy scores were higher in participants who felt less pleasure in daily life, were less dynamic and suffered from their isolation. They were also socially inhibited and did not perform leisure activities.

*Psychopathology* Correlation analyses showed that executive apathy scores were strongly positively correlated with depressive symptom score on BDI (r = 0.548; p < 0.001; 95%confidence interval CI[0.521,0.573]) and negatively correlated with self-esteem score (SES) (r = − 0.489; p < 0.001; 95%CI[− 0.516,− 0.460]).

ANOVAs on executive apathy scores revealed significant, large effects of level of subjective fatigue (F(3,2785) = 195; p < 0.001; η^2^ = 0.174), moderate effects of self-perceived anxiety (F(1,2787) = 215; p < 0.001; η^2^ = 0.072) and psychiatric disorder (F(1,2787) = 171; p < 0.001, η^2^ = 0.058) and small effects of use of psychotropics (F(1,2787) = 53.6; p < 0.001; η^2^ = 0.019), presence of psychiatric disorders in first-degree relatives (F(1,2787) = 41.2; p < 0.001; η^2^ = 0.015) and somatic disorder (F(1,2787) = 40.7; p < 0.001; η^2^ = 0.014). Thus, executive scores were higher in respondents who were exhausted, anxious, had a psychiatric disorder, used psychotropics, had a first-degree relative with psychiatric disorders, or had a somatic disorder themselves.

##### Emotional apathy

*Sociodemographic* ANOVAs on emotional apathy scores revealed that gender was the only sociodemographic variable to have a large, significant effect (F(2,2786) = 218; p < 0.001; η^2^ = 0.135). Bonferroni tests revealed that men had higher scores than women (t(2786) = 20.85; p < 0.001; d = 0.39), while there was no difference between men and trans individuals or between women and trans individuals.

*Education* ANOVAs on emotional apathy scores revealed significant, small effects of type of field (F(4,2784) = 8.07; p < 0.001; η^2^ = 0.010) and of level of study (F(5,2783) = 6.21; p < 0.001; η^2^ = 0.010). More precisely, Bonferroni tests showed that emotional apathy scores were higher in the technical and sciences field than the other fields (t(2784) = 5.08; p < 0.001; d = 0.09) and for first years than Master’s degree students (t(2783) = 3.71; p < 0.02; d = 0.07).

*General functioning* ANOVAs on emotional apathy scores revealed small, significant effects of isolation (F(2,2786) = 39.1; p < 0.001; η^2^ = 0.027), daily pleasure (F(3,2785) = 17.6; p < 0.001; η^2^ = 0.019), number of social contacts initiated (F(3,2785) = 17.2; p < 0.001; η^2^ = 0.018) and dynamism (F(1,2787) = 49.3; p < 0.001; η^2^ = 0.017). Indeed, emotional apathy scores were higher in participants who did not suffer from their isolation, felt less pleasure in daily life and were also socially inhibited and less dynamic.

*Psychopathology* Correlation analyses showed that emotional apathy scores were negatively correlated with anticipatory pleasure (r = − 0.340; p < 0.001; 95%CI[− 0.372,− 0.306]) and consummatory pleasure (r = − 0.257; p < 0.001; 95%CI[− 0.292,− 0.222]), measured by the Temporal Experience of Pleasure Scale (TEPS).

ANOVAs on emotional apathy scores revealed significant, small effects of anxiety (F(1,2787) = 27.9; p < 0.001; η^2^ = 0.010). Indeed, emotional apathy scores were higher in respondents who were less anxious.

##### Initiative apathy

*Sociodemographic* ANOVAs on initiative apathy scores revealed that gender was the only sociodemographic variable to have a significant, small effect (F(2,2786) = 13.7; p < 0.001; η^2^ = 0.010). Bonferroni tests revealed that men had higher scores than women (t(2786) = 4.89; p < 0.001; d = 0.10), while there was no difference between men and trans individuals or between women and trans individuals.

*Education* ANOVAs performed on scores of initiative apathy revealed a significant, small effect of honours at Bachelor degree level (F(5,2783) = 13.0; p < 0.001; η^2^ = 0.023), type of field (F(4,2784) = 15.0; p < 0.001; η^2^ = 0.021), choice of field (F(3,2785) = 17.7; p < 0.001; η^2^ = 0.019) and number of repeated class (F(7,2781) = 5.90; p < 0.001; η^2^ = 0.015). More precisely, Bonferroni tests showed that initiative apathy scores were higher for students without than with honours on their Bachelor’s degree (t(2783) = 6.12; p < 0.001; d = 0.11), for students in the arts and literature field rather than in law or health (t(2786) = 5.55; p < 0.001; d = 0.10), for students in a field they had chosen but did not like compared to those who had chosen their field and liked it (t(2785) = 7.01; p < 0.001; d = 0.13) and for students that had repeated three class rather than none (t(2781) = 4.32; p < 0.001; d = 0.08).

*General functioning* ANOVAs on initiative apathy scores revealed significant, large effects of dynamism (F(1,2787) = 803; p < 0.001; η^2^ = 0.224), daily pleasure (F(3,2785) = 194; p < 0.001; η^2^ = 0.173) and number of initiated social contacts (F(3,2785) = 124; p < 0.001; η^2^ = 0.118); moderate effects of isolation (F(2,2786) = 127; p < 0.001; η^2^ = 0.083) and frequency of social evenings (F(3,2785) = 52.3; p < 0.001; η^2^ = 0.053) and small effects of leisure (F(1,2787) = 100; p < 0.001; η^2^ = 0.035), curiosity (F(1,2787) = 92.5; p < 0.001; η^2^ = 0.032), independence (F(1,2787) = 35.1; p < 0.001; η^2^ = 0.012) and frequency of household cleaning (F(1,2787) = 34.5; p < 0.001; η^2^ = 0.012). Indeed, initiative apathy scores were higher in participants who were less dynamic, felt less pleasure in daily life, were socially inhibited and isolated. They also had fewer social engagements, did not perform leisure activities, were less curious, were more dependent and cleaned their home less frequently.

*Psychopathology* Correlation analyses showed that initiative apathy scores were positively correlated with depressive symptoms (BDI) (r = 0.401; p < 0.001; 95%CI[0.370,0.432]) and negatively correlated with self-esteem (SES) (r = − 0.396; p < 0.001; 95%CI[− 0.427,− 0.365]), anticipatory pleasure (TEPS) (r = − 0.366; p < 0.001; 95%CI[− 0.398,− 0.333]) and consummatory pleasure (TEPS) (r = − 0.254; p < 0.001; 95%CI[− 0.279,− 0.210]).

ANOVAs on initiative apathy scores revealed significant, moderate effects of fatigue (F(3,2785) = 47.2; p < 0.001; η^2^ = 0.048) and small effects of anxiety (F(1,2787) = 64.3; p < 0.001; η^2^ = 0.023) and psychiatric disorder (F(1,2787) = 51.6; p < 0.001; η^2^ = 0.018). Indeed, initiative apathy scores were higher in respondents who felt exhausted, felt anxious and had a psychiatric disorder.

#### Identification of the predictors of each form of apathy

**Figure 3:**
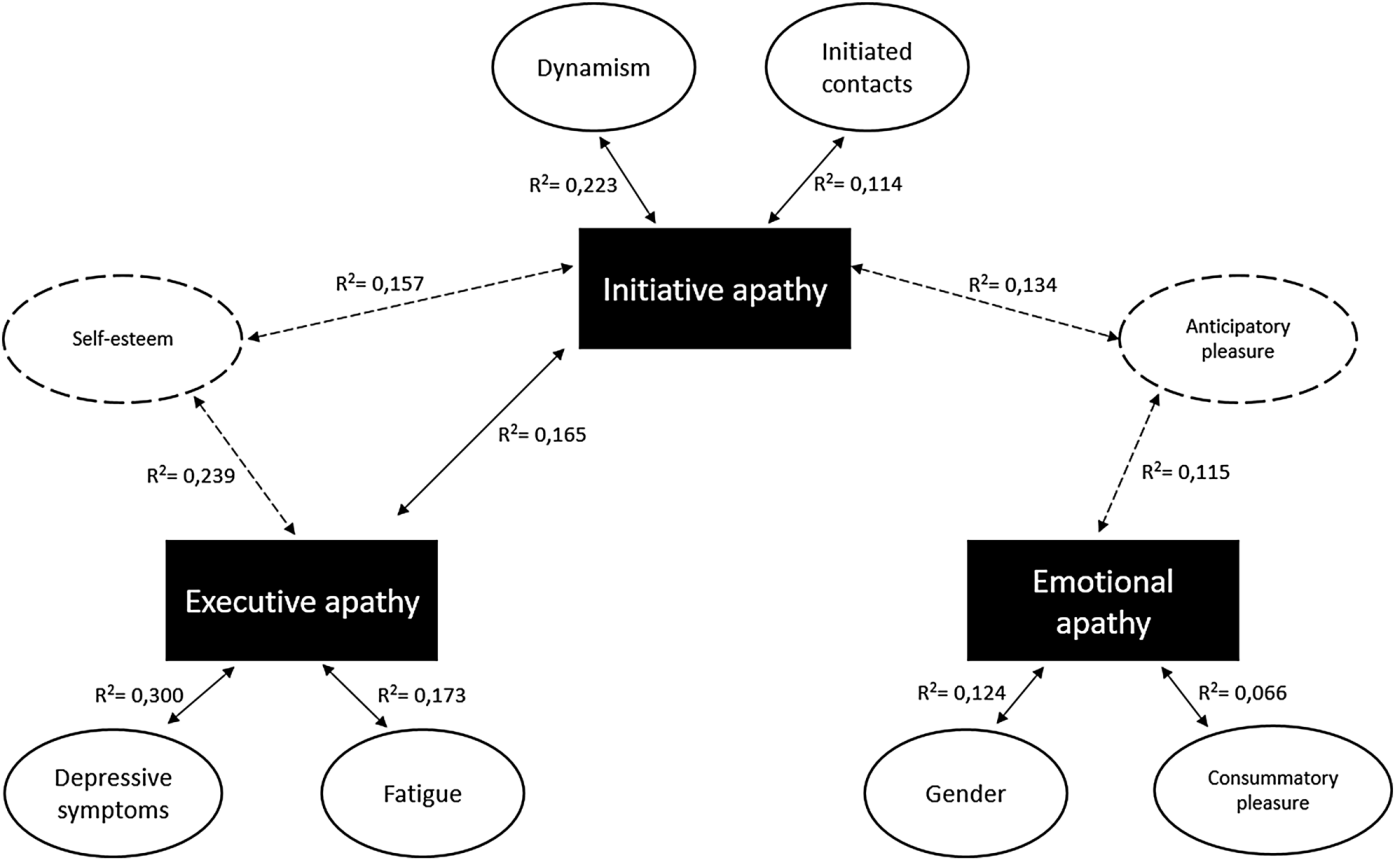
Predictors for the three forms of apathy.

Figure 3 summarizes survey variables that best predicted each form of apathy.

In a linear regression model with executive DAS score as the dependent variable, depression (B = 0.210; SE = 0.018; 95%CI[0.175,0.246]; β = 0.284; t = 11.58; p < 0.001), initiative apathy (B = 0.252; SE = 0.020; 95%CI[0.212,0.291]; β = 0.208; t = 12.50; p < 0.001), fatigue (B = 1.038; SE = 0.109; 95%CI[0.822,1.253]; β = 0.168; t = 9.45; p < 0.001) and self-esteem (B = − 0.093; SE = 0.017; 95%CI[− 0.128,− 0.058]; β = − 0.121; t = − 5.26; p < 0.001) explained 37.0% of the variance in executive apathy scores (F(4,2784) = 407; p < 0.001; AIC = 15,356).

In a linear regression model with emotional DAS score as the dependent variable, gender (B = − 2.737; SE = 0.161; 95%CI[− 3.052,− 2.422]; β = − 0.292; t = − 17.02; p < 0.001), anticipatory pleasure (B = − 0.135; SE = 0.011; 95%CI[− 0.157, − 0.114]; β = − 0.234; t = − 12.54; p < 0.001) and consummatory pleasure (B = − 0.084; SE = 0.012; 95%CI[− 0.109, − 0.059]; β = − 0.124; t = − 6.73; p < 0.001) explained 21.3% of the variance in emotional apathy scores (F(3,2785) = 253; p < 0.001; AIC = 15,284).

In a linear regression model with initiative DAS score as the dependent variable, dynamism (B = − 2.159; SE = 0.145; 95%CI[− 2.445,− 1.873]; β = − 0.548; t = − 14.81; p < 0.001), executive apathy (B = 0.194; SE = 0.014; 95%CI[0.166,0.222]; β = 0.235; t = 13.61; p < 0.001), self-esteem (B = − 0.059; SE = 0.011; 95%CI[− 0.081,− 0.036]; β = − 0.093; t = − 5.15; p < 0.001), anticipatory pleasure (B = − 0.119; SE = 0.008; 95%CI[− 0.136, − 0.103]; β = − 0.221; t = − 14.08; p < 0.001) and number of contacts initiated (B = − 0.821; SE = 0.076; 95%CI[− 0.970,− 0.673]; β = − 0.169; t = − 10.82; p < 0.001) explained 39.0% of the variance in initiative apathy scores (F(5,2783) = 356; p < 0.001; AIC = 14,198).

### Study 2

#### Confirmation of the presence of the three forms of apathy

Of the respondents, 26.5% had an executive apathy trait (cut-off:16), 19.4% an emotional apathy trait (cut-off:12) and 18.4% an initiative apathy trait (cut-off:14); 5.6% had an executive apathy symptom (cut-off:21), 5.8% an emotional apathy symptom (cut-off:16) and 4.1% an initiative apathy symptom (cut-off:18).

**Figure 4:**
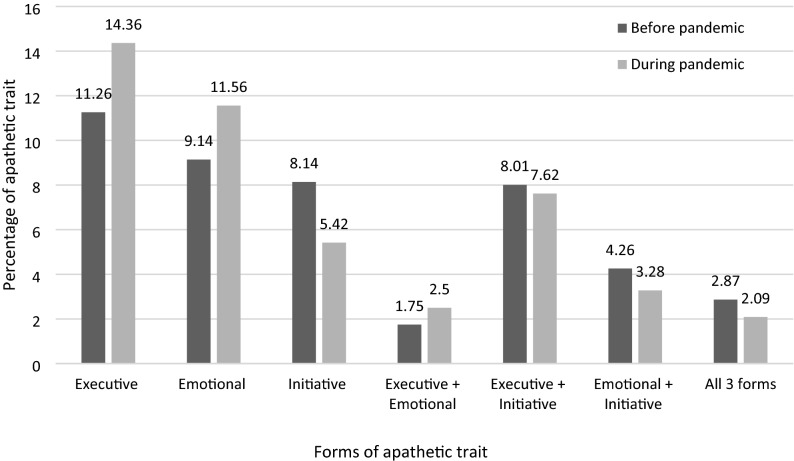
Comparison of combinations of multidimensional apathetic trait in first-year students between before and during the COVID-19 pandemic.

Specifically, 31.34% of respondents presented a single form of apathetic trait (14.36% executive, 11.56% emotional and 5.42% initiative apathy), 13.41% combined two forms, and 2.09% had all three forms. Figure 4 illustrates a comparison of the percentage of combinations for the three forms of apathetic trait in first-years students, in both studies.

Among the respondents, 12.22% presented a single form of apathetic symptom (4.41% executive, 5.24% emotional and 2.56% initiative apathy), 1.66% had mixed symptoms (0.18% executive and emotional forms, 1.07% executive and initiative forms, and 0.42% emotional and initiative forms), and none (0.00%) presented all three symptoms together.

Analyses of correlations between the three forms of subclinical apathy showed that executive and emotional apathy were still not correlated in the second study (r = − 0.037; p > 0.05), whereas initiative apathy was still positively correlated with both executive (r = 0.459; p < 0.001) and emotional apathy (r = 0.181; p < 0.001).

#### Interests of two main predictors: gender and depression

ANOVAs on executive and initiative apathy scores revealed that gender had no significant effect. ANOVAs on emotional apathy scores revealed that gender had a medium, significant effect (F(1,1676) = 161; p < 0.001; η^2^ = 0.088). Bonferroni tests revealed that men had higher scores than women (1676) = 12.7; p < 0.001; d = 0.31).

Correlation analyses showed that depressive symptom score on the BDI were positively correlated with executive apathy scores (r = 0.286; p < 0.001; 95%CI[0.242,0.330]) and initiative apathy scores (r = 0.178; p < 0.001; 95%CI[0.131,0.224]).

In a linear regression model with executive DAS score as the dependent variable, initiative apathy (B = 0.515; SE = 0.026; 95%CI[0.464,0.566]; β = 0.422; t = 19.70; p < 0.001) and depression (B = 0.423; SE = 0.043; 95%CI[0.339,0.507]; β = 0.211; t = 9.86; p < 0.001) explained 25.5% of the variance in executive apathy scores (F(2,1675) = 286; p < 0.001; AIC = 9720).

In a linear regression model with emotional DAS score as the dependent variable, gender (B = − 2.585; SE = 0.206; 95%CI[− 2.988,− 2.182]; β = − 0.594; t = − 12.58; p < 0.001) and initiative apathy (B = 0.178; SE = 0.024; 95%CI[0.130,0.225]; β = 0.169; t = 7.35; p < 0.001) explained 11.6% of the variance in emotional apathy scores (F(2,1675) = 110; p < 0.001; AIC = 9499).

In a linear regression model with initiative DAS score as the dependent variable, executive apathy (B = 0.383; SE = 0.017; 95%CI[0.349,0.417]; β = 0.467; t = 22.08; p < 0.001) and emotional apathy (B = 0.189; SE = 0.020; 95%CI[0.150,0.229]; β = 0.199; t = 9.38; p < 0.001) explained 25.0% of the variance in initiative apathy scores (F(2,1675) = 280; p < 0.001; AIC = 9062).

#### Stability of the three forms of apathy

For first-year students, Table 2 presents a comparison of the scores of the three forms of subclinical apathy and depressive symptoms, in 2020 and 2021. Only BDI–II score showed a large significant difference in the new cohort tested 1 year later, with an increase of 5.37 points (BDI 2021–2020 = 14.52–9.15).

**Table 2:** Comparison of scores of the validated questionnaires for first-year students, in 2020 and 2021.

Questionnaire	February 2020		February 2021		Stats
	Mean ± standard deviation	Minimum–maximum	Mean ± standard deviation	Minimum–maximum	
DAS total	29.89 ± 9.11	8–64	29.64 ± 9.28	7–60	Student’s t(2475) = 0.63; p = 0.528
					Cohen’s d = 0.03; 95% CI [− 0.057;0.111]
DAS executive	11.86 ± 4.75	1–23	12.14 ± 5.07	0–24	Welch’s t(1663) = − 1.34; p = 0.180
					Cohen’s d = − 0.06; 95% CI [− 0.141;0.028]
DAS emotional	7.65 ± 4.31	0–24	7.79 ± 4.35	0–24	Student’s t(2475) = − 0.786; p = 0.432
					Cohen’s d = − 0.03; 95% CI [− 0.118;0.050]
DAS initiative	10.38 ± 4.10	0–24	9.71 ± 4.15	0–24	Student’s t(2475) = 3.81; p < 0.01
					Cohen’s d = 0.164; 95% CI [0.078;0.248]
BDI–II	9.15 ± 6.80	0–38	14.52 ± 2.53	2–36	Welch’s t(905) = − 21.6; p < 0.001
					Cohen’s d = − 1.22; 95% CI [− 1.33;− 1.12]

*DAS* The Dimensional Apathy Scale (Radakovic and Abrahams^47^), *BDI-II* The Beck Depression Inventory II (Beck et al.^52^).

## Discussion

The results revealed the presence and stability of the three forms of apathetic trait in students over a year and identified form-specific determinants, confirming their independence. In addition, by revealing for the first time a specific link between one form of apathy (the executive form) and depressive symptoms, they shed new light on the overlap between apathy and depression that continues to cause confusion. Taken together, the present results open new avenues to explore to develop new, targeted therapeutic treatments.

### Subclinical apathy was present in three distinct forms in a student population and associated with the same debilitating consequences as apathetic symptom

The present investigation, based on two studies involving, respectively, 2789 and 1678 students, confirmed the presence of subclinical apathy in three distinct forms in a student population. About 40% of respondents presented an apathetic trait, in agreement with the 35% found in the only other study in a similar population^11^. About 10–14% of respondents presented an apathetic symptom, which was far above the 2% found in other studies with young people^9^. However, in these studies, no distinction was made between the three forms of apathy and the scales used were different: 2% may correspond to participants with the most severe form of apathy (i.e., initiative apathy).

An exploration of factors related to education and general functioning clearly revealed, in agreement with some other studies, that subclinical apathy, of whichever form, was associated with debilitating consequences in academic and daily life^13^. Initiative apathy was the most debilitating form in daily life, and emotional apathy specifically in social life. As previously shown, all negative repercussions were similar, although perhaps differing in intensity, to those of the apathetic symptom^19,30^. The present results are thus completely consistent with the usual definition of apathy and support keeping the overarching term “apathy” for all three forms, despite their not having the same underlying mechanisms.

Regarding the population of students, our results show that the dominant form of apathy differed according to field of study, suggesting that course choices may be related to the difficulties associated with the specific form of apathy affecting a student. Students with executive or initiative apathy were more often studying art and literature whereas students with emotional apathy more often studied technical subjects and sciences. This question, as yet unexplored, could be an interesting object of investigation for educational scientists.

### From the independence of the three forms of apathy to a better characterization of each

Our results provide strong evidence for the independence of the three forms of apathy. This is because they highlight that the majority of participants with a subclinical apathy presented a single form of apathy. They also show the existence of specific correlates or predictors for each of the three forms. Of the respondents with apathetic trait, 10–15% had only executive apathy, 10% only emotional apathy and 5–8% only initiative apathy. We expected a lower prevalence of the third form in a young healthy population because it is the most severe form, associated with greater disability^31^.

Most factors specifically linked to a form of apathy concerned psychopathology. Regarding executive apathy, our results suggest that it may be a secondary form. It was the only form specifically linked to psychiatric or somatic disorders and the only one to be predicted by depressive symptoms and fatigue. Executive apathy may therefore be secondary to another disorder, potentially modulated by executive deficits. In the literature, executive apathy has been linked to deficits in executive functions (Alzheimer: Perri et al.^25^; Schizophrenia: Raffard et al.^26^), executive deficits which also correlate with depression^32,33^ and fatigue^34,35^. These results cast doubt on the nature of executive apathy, but also shed light on the confusion between apathetic and depressive symptoms, suggesting that the overlap may be specific to the executive form.

Emotional apathy, in contrast, was the only form to be predicted by the two motivational components: consummatory and anticipatory pleasure. However, only consummatory pleasure was specific to emotional apathy; anticipatory pleasure also predicted the initiative apathy. Emotional apathy was therefore the only form linked to a deficit of hedonic pleasure in the moment. To our knowledge, the only link previously highlighted between apathy and motivation was non-specific: a link between deficit of anticipatory pleasure and one-dimensional apathy^36^. Our results revealed the importance of considering all the components of motivation to improve understanding of apathy in its multidimensional form.

Finally, and interestingly, our results may suggest the presence of two subtypes of initiative apathy: a mixed form with both executive and emotional deficits, and a specific deficit. Initiative apathy was correlated with the two other forms of apathy over 2 years. Moreover, in 60–70% of cases, initiative apathy was present in association with another form. This mixed form hypothesis is also supported by neuroanatomical and clinical studies in patients with neurodegenerative disease, especially Parkinson’s and Alzheimer’s^25,37,38^. These studies showed a combination of cognitive and emotional deficits and the presence of lesions characteristic of the two other forms. The second subtype of initiative apathy could be a pure deficit in action initiation, the last step before action is taken. Although the percentage was low, the fact remains that 5–8% of our sample had initiative apathy without any other associated form. Such pure initiative apathy has previously been highlighted in amyotrophic lateral sclerosis, confirming the existence of this pure form as a symptom^39,40^ related to specific cerebral regions (anterior cingulate cortex and supplementary motor area) in patients^24,41^ and in healthy people^31,42^.

### The stability of subclinical apathy and the effects of the COVID-19 pandemic

The three dimensions of subclinical apathy were stable under different circumstances. Indeed, before and during a global pandemic, there were no major variations of means, standard variations, correlates, predictors and cut-off for apathetic trait and symptom. Only initiative apathy as a pure deficit of action initiation showed a slight decrease in the second study. However, social distancing and lockdown could explain this reduction, by changed social norms and reduced everyday activities for everyone.

Furthermore, the stability of apathy contrasts with the sharp increase in depression. One year later, depression was still a specific predictor of executive apathy. However, in the second study, the rise of depression during the COVID-19 pandemic allowed us to improve our knowledge of the link between executive apathy and depression. Depressive symptoms doubled over 1 year in students, probably because of lockdowns, curfews and psychological difficulties induced by the COVID-19 pandemic^43,44^, but executive apathy remained stable. Thus, even if depressive symptoms are a strong predictor of executive apathy, it appears that executive apathy remains a separate symptom with other explanatory factors.

### What role do gender and self-esteem play in apathy?

To our knowledge, no other studies explored the link between gender and a specific form of apathy. However, others have revealed a positive correlation between male gender and one-dimensional apathy^30,45^. Our results showed more precisely that men presented more emotional apathy than women and trans individuals whereas transgender experienced more executive apathy than cisgender (male and female). The male gender was still a predictive factor of emotional apathy 1 year later. Although no real explanation for this association is yet apparent, controlling for gender seems important for studying apathy.

The identification of self-esteem as a predictor for executive and initiative apathy may offer new perspectives for non-pharmacological therapeutic interventions focused on enhancing self-esteem when these two forms of apathy are present. Two previous studies, involving patients with traumatic brain injury^41^ and psychotic disorders^36^, showed that low self-esteem, by inducing demotivating beliefs, may prevent people from undertaking new or complex tasks, thereby inducing apathy.

## Conclusion

This study confirmed the existence and stability of multidimensional subclinical apathy in young healthy students under different circumstances (global pandemic vs not) and showed that apathetic trait was associated with the same debilitating consequences in all spheres of daily life as apathetic symptom. By identifying specific characteristics for three forms of apathy, the present results may aid exploration of the mechanisms underlying multidimensional apathy (especially self-esteem, anticipation, and motivation) in healthy people and in various pathologies to propose new, targeted therapies.

## Methods

The current investigation, based on two studies, aims to address the incidence of multidimensional apathetic trait in three distinct forms (executive, emotional and initiative) in a student population, to specify its determinants and to evaluate its stability during a global pandemic. Two online surveys, conducted 1 year apart, tested 2789 and 1678 different university students, with qualitative measures and validated scales.

### Participants

Participants were recruited in a French University. All registered students received an email giving them the opportunity to participate in an online survey about ‘Personality in university students’, created on LimeSurvey. To participate, the only criteria were to be a student, aged between 18 and 28 years and to be a native French-speaker. All participants gave electronic informed consent, and the study was approved by the University review board (UNISTRA/CER/2020-13) and the French data protection authority (CNIL). They weren’t paid for their participation.

In total, for study 1, 3144 students (26.5% male) completed the survey. After eliminating 355 respondents who did not match the criteria, 2789 students constituted the study sample. For study 2, 1678 new first-year students (38.6% male) constituted the study sample (see Table 3 for sociodemographic characteristics of the studies).

**Table 3:** Sociodemographic characteristics of the sample.

	Study 1 (2020)	Study 2 (2021)
Number	2789 participants	1678 participants
Gender	729 males (26.1%), 2040 females (73.1%), 20 trans individuals (0.7%)	648 males (38.6%), 1030 females (61.4%)
Age	20.8 years (± 2.2); range 18–28	19.2 years (± 1.4); range 18–22
Level of study	First year: 799 (28.6%)	First year: 1678 (100%)
	Second year: 596 (21.4%)	
	Third year: 460 (16.5%)	
	Fourth year: 483 (17.3%)	
	Fifth year: 338 (12.1%)	
	PhD: 113 (4.1%)	
Field of study	Arts, literature, languages: 382 (13.7%)	–
	Law, economy, politics: 599 (21.5%)	
	Health: 434 (15.6%)	
	Humanities and social: 728 (26.1%)	
	Technological sciences: 646 (23.2%)	

### Online survey

For Study 1, the survey was divided in four parts: sociodemographic characteristics, education, general functioning, and psychopathology (see Supplementary data 2 online). All these factors were assessed with qualitative measures. Four validated scales were added to complete the assessment of psychopathology. The names and themes of the four questionnaires were not specified online.

1. The Dimensional Apathy Scale (DAS)^47^ is a 24-item scale assessing the severity of the 3 forms of apathy (Executive, Emotional and Initiative apathy). Items are scored on a 4-point Likert scale based on the frequency of occurrence of the apathetic symptoms in the previous month. 3 scores are obtained, one for each form of apathy (based on 8 items each). A high score (maximum, 24) indicates a severe form of apathy.
2. The Beck Depression Inventory II (BDI-II) (Beck et al.^52^) is a 13-item scale assessing the severity of depressive symptoms. Each item is score from 0 to 3. A high global score (maximum, 39) indicates severe depression.
3. The Rosenberg Self-Esteem Scale (RSE) (Rosenberg^53^) is a 10-item scale assessing self-esteem. Each item is score from 1 to 4. A low global score (minimum, 10) indicates severe self-esteem disorder.
4. The Temporal Experience of Pleasure Scale (TEPS) (Gard et al. 2006) is an 18-item scale assessing anticipatory (10 items) and consummatory (8 items) pleasure. Each item is score from 1 to 6. Anticipatory pleasure refers to pleasure due to active search for or anticipation of a pleasant activity (score range: 10 to 60). Consummatory pleasure refers to pleasure directly due to accomplishing a pleasant activity (score range: 8 to 48). A low score for each subscale indicates pleasure disorder^46^.

For study 2, the survey was divided into two parts: sociodemographic characteristics and psychopathology. Two validated questionnaires were used to assess the psychopathology: the DAS^47^ and the BDI-II (Beck et al.^52^).

See Supplementary data 3 online for correlations between all the questionnaires for both studies.

### Statistical analysis

Each participant had three DAS subscores: one for each form of apathy.

To determine the percentage of participants with a subclinical apathy, a cut-off was calculated for each subscore, based on the mean and the standard deviation of studies samples (Study 1, 2789 students, Study 2, 1678 first-year students). The suggested cut-offs were + 1 standard deviation from the mean for subclinical apathy/apathetic trait and + 2 standard deviation from the mean for clinical apathy / apathetic symptom^11,39,48^.

Both studies had large sample size, so according to the Central Limit Theorem, data could be approximated by a normal distribution and parametric tests were used^49^.

Pearson correlation tests were implemented between DAS scores.

To identify variables correlating with each form of apathy, Pearson correlation tests were implemented with continuous variables; ANOVAs and Bonferroni post-hoc tests were used with categorical variables.

Exploratory approach was used for regressions analyses since the selection of predictor variables was not based on a priori hypotheses. Predictors of each form of apathy were specified by multiple linear regression analyses, using one DAS score as reference and all the other variables from the online survey as possible regressors of interest (see Supplementary data 4 online). The purpose was to determine which variables in the survey most influenced each form of apathy, thanks to the Akaike Information Criterion (AIC). The smaller the AIC value, the better the model fit, using the fewest possible variables. R-square values (the percentage variation of the reference explained by the regressor of interest) were calculated.

In study 2, to compare questionnaire scores across time, Student’s t test was used when there was an assumption of equal variances with Levene’s test (p > 0.05); Welch’s t test was used when there was a violation of the assumption of equal variances with Levene’s test (p < 0.05).

The statistical significance level was set at 0.05. As both studies have a large sample size, effect size measures were always added, to prevent big data bias and to analyze only practical effects^50,51^. For correlation squared (R^2^ and η^2^) used in regressions and ANOVA, practical effect was small around 0.01, medium around 0.06 and large from 0.14. For correlation coefficient (r) used in correlations, practical effect was small around 0.10, medium around 0.30 and large from 0.50. For standardized mean difference (d) used in t-test, practical effect was small around 0.20, medium around 0.50 and large from 0.80. Only practical significant results are described in the Results section below.

### Ethical standards

The authors assert that all procedures contributing to this work comply with the ethical standards of the relevant national and institutional committees on human experimentation (Research ethics committee of the University of Strasbourg—UNISTRA/CER/2020-13) and with the Helsinki Declaration of 1975, as revised in 2008.

## Supplementary Information

Supplementary Information.

## Data Availability

The datasets generated during and/or analyzed during the current study are available from the corresponding author on reasonable request.
