# Author Correction: The stability of multidimensional subclinical apathy during a pandemic and its relations to psycho-behavioral factors

**DOI:** 10.1038/s41598-022-08514-4

**Published:** 2022-03-14

**Authors:** Giulia Lafond-Brina, Anne Bonnefond

**Affiliations:** 1 INSERM U1114, Pôle de Psychiatrie, 1 Place de l’Hôpital, 67000 Strasbourg, France; 2 University of Strasbourg, Strasbourg, France

Correction to: *Scientific Reports* 10.1038/s41598-022-06777-5, published on 21 February 2022

The original version of this Article contained an error in the spelling of the authors Giulia Lafond-Brina and Anne Bonnefond which were incorrectly given as Lafond-Brina Giulia and Bonnefond Anne.

The original Article has been corrected.

