# Supplementary material for: The stability of multidimensional subclinical apathy during a pandemic and its relations to psycho-behavioral factors
Source: Sci Rep. 2022 Feb 21;12:2931. doi: 10.1038/s41598-022-06777-5 (PMC8860996; doi:10.1038/s41598-022-06777-5)
Supplement: Supplementary file 1 — Supplementary Information. [file 41598_2022_6777_MOESM1_ESM.pdf]

The stability of multidimensional subclinical apathy during a pandemic and its relations to psycho-behavioral factors

Supplementary data 1-a: Distribution of the three DAS scores in the sample, with cut-offs for subclinical (trait) and clinical (symptom) apathy

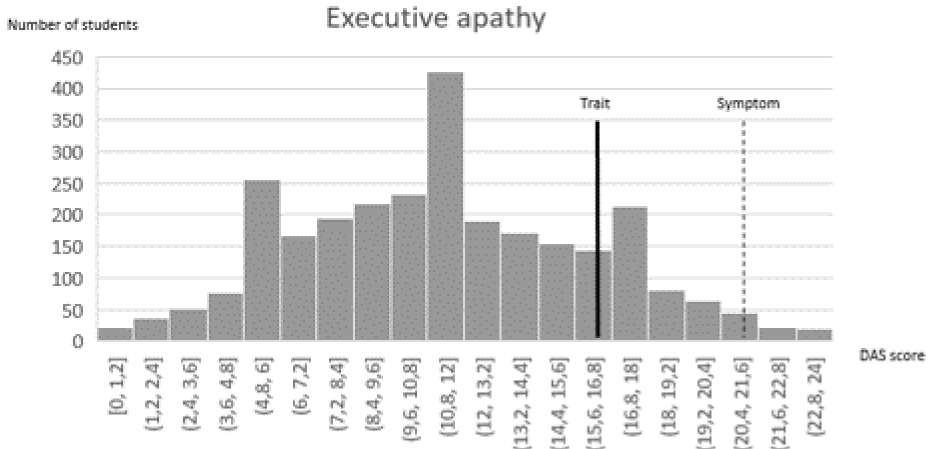

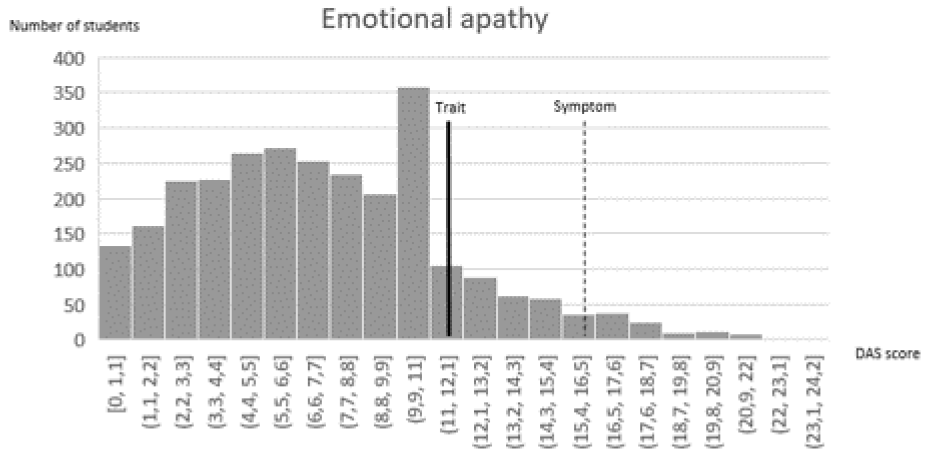

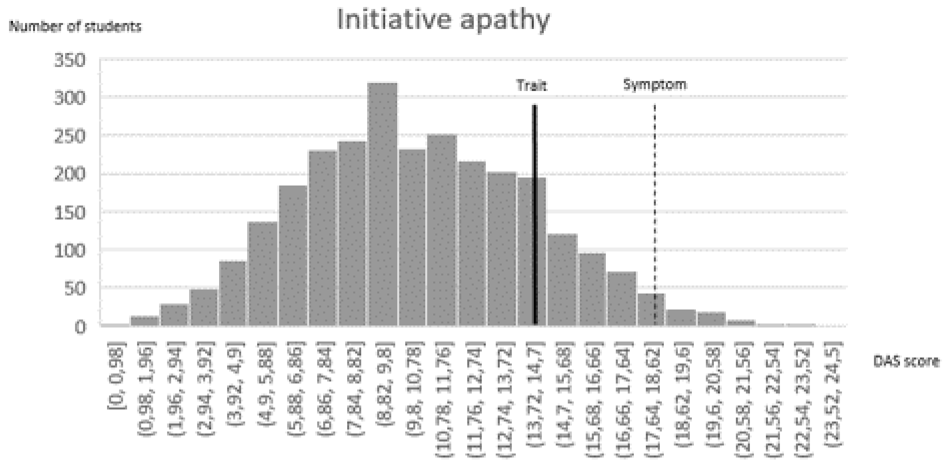

Supplementary data 1-b: Table of the three DAS cut-offs for subclinical (trait) and clinical (symptom) apathy in Study 1 and Study 2

	Cut-off for subclinical apathy		Cut-off for clinical apathy	
	Study 1	Study 2	Study 1	Study 2
DAS executive	16	16	21	21
DAS emotional	12	12	16	16
DAS initiative	14	14	18	18

Supplementary data 2: Online survey

1- Sociodemographic characteristics

Gender: ☐ male ☐ female ☐ neutral

Age? : ………………………………….

Is French your mother tongue? ☐ Yes ☐ No

2- Education

Are you currently enrolled at the University of Strasbourg? ☐ Yes ☐ No

In which field? ………………………………….

In the event of a double course, indicate your main course

In which level?

- First year
- Diploma of Higher Education
- BA, BS/BSc
- MS/MSc, MA
- Master's Degree
- PhD

What were your results in the baccalaureate?

- Mention very well with congratulations from the jury
- Honours
- Mention well
- Mention good enough
- Without honours
- I do not remember

Did you repeat a year during your schooling?

- No
- Yes
  - in kindergarten
  - in elementary school
  - in middle-school
  - in high school
  - at university

If so, how many times so far? ………………………………….

Did you skip a class during your schooling?

- No
- Yes
    - in kindergarten
    - in elementary school
    - in middle-school
    - in high school
    - at university

If so, how many times so far? ………………………………….

Did you choose your field this year?

- Yes and I like what I study
- Yes but, in the end, I don't like what I'm studying
- No and I don't like what I'm studying
- No, but in the end, I like what I study

3- General functioning

On average, how many people do you initiate social contact with in a day:

- 0
- 1 to 2
- 3 to 6
- More than 6

Face to face, phone calls or messages are considered social contacts.

Would you say you are socially isolated?

- Yes and I suffer from it
- Yes but it’s ok for me
- No

Do you have any hobbies? : ☐ Yes ☐ No

If so, list them:

Do you go out at night?

- Almost every evening
- Often (several evenings per week)
- Rarely (a few evenings per month)
- Almost never

Do you like to discover new things? ☐ Yes ☐ No

Would you describe yourself as a dynamic and enthusiastic person in your daily life?

☐ Yes ☐ No

Do you feel pleasure in your daily life?

- Almost all the time
- Often
- Rarely
- Almost never

Do you live?

- Alone in an apartment
- Alone in a dormitory
- With your family
- With a roommate
- With a partner
- In a guest room
- No fixed address

Do you consider yourself independent on a daily basis? ☐ Yes ☐ No

Do you do the housework? ☐ Yes ☐ No

Do you do your grocery shopping? ☐ Yes ☐ No

Do you cook your meals?

- Yes, lunch and dinner
- Yes, lunch or dinner
- No, I eat at the (university) restaurant
- No, someone makes me my meals

How much time do you spend sitting or lying down on a typical day?

Indicate the approximate number of hours: ………………………………….

This question includes time spent sitting at a desk, traveling in car, bus, train, reading, playing cards or watching TV. <u>It does not include the time spent asleep</u>.

4- Psychopathology

Qualitative measures

Do you currently suffer from a somatic disease? ☐ Yes ☐ No

For example, are taken into account for this question: a motor or sensory disability, a thyroid disorder, a sleep disorder, a neurological disease,

Do you have a neurodevelopmental disorder? ☐ Yes ☐ No

For example: ADHD, dyslexia, dyspraxia …

If so, specify the neurodevelopmental disorder(s): ………………………………….

Do you suffer or have you ever suffered from a psychiatric disorder or a mood disorder?

☐ Yes ☐ No

For example: depression, bipolarity, schizophrenia …

Do your parents or siblings suffer or have they suffered from a psychiatric disorder or a mood disorder? ☐ Yes ☐ No

Have you taken any psychotropic drugs in the past three months? ☐ Yes ☐ No

By psychotropic drugs are meant: antidepressants, anxiolytics, sleeping pills, antipsychotics and mood regulators

Do you take any of the following substances?

- Alcohol : ☐ Yes ☐ No
  If so, number of drinks per week: …………………………………
- Cannabis : ☐ Yes ☐ No
  If so, how many times per month: ………………………………….

  Was the last intake in the past month? ☐ Yes ☐ No
- Other illicit drugs : ☐ Yes ☐ No
  Was the last intake in the past month? ☐ Yes ☐ No

Do you feel tired every day <u>for no reason?</u>

- Always
- Often (several times per week)
- Rarely (once a week, or not every week)
- Never

Are you worried about your future? ☐ Yes ☐ No

Validated scales

<u>Scale 1</u>: The Rosenberg Self-Esteem Scale (RSE) (Rosenberg, 1965)

<u>Scale 2</u> : Dimensional Apathy Scale (Radakovic & Abrahams, 2014)

<u>Scale 3</u>: The Temporal Experience of Pleasure Scale (TEPS) (Gard and al, 2006)

<u>Scale 4</u>: The Beck Depression Inventory II (BDI-II) (Beck et al., 1996)

Supplementary data 3: Correlations between all the questionnaires for both studies

Study 1						
	DAS emotional	DAS initiative	BDI-II	RSE	TEPS anticipatory	TEPS consummatory
DAS executive	r=-0.06 p>.05	r=0,40 p<.001	r=0,55 p<.001	r=-0,49 p<.001	r=-0,08 p<.001	r=-0.01 p>.05
DAS emotional		r=0,20 p<.001	r=-0,01 p>.05	r=0,08 p<.001	r=-0,34 p<.001	r=-0,26 p<.001
DAS initiative			r=0,40 p<.001	r=-0,39 p<.001	r=-0,36 p<.001	r=-0,25 p<.001
BDI-II				r=-0,78, p<.001	r=-0,26 p<.001	r=-0,13 p<.001
RSE					r=0,22 p<.001	r=0,10 p<.001
TEPS anticipatory						r=0,41 p<.001
Study 2						
	DAS emotional	DAS initiative	BDI-II			
DAS executive	r=-0,04 p>.05	r=0,46 p<.001	r=0,28 p<.001			
DAS emotional		r=0,18 p<.001	r=0,02 p>.05			
DAS initiative			r=0,18 p<.001			

DAS - The Dimensional Apathy Scale (Radakovic and Abrahams, 2014) ; BDI-II - The Beck Depression Inventory II (Beck et al., 1996) ; RSE - The Rosenberg Self-Esteem Scale (Rosenberg, 1965) ; TEPS - The Temporal Experience of Pleasure Scale (Gard and al, 2006)

Supplementary data 4: Independent variables and their modalities

	Independent variables	Modalities if categorical variables
**Sociodemographic characteristics**	Gender	3: Male; Female; Transgender
	Age	Years
**Education**	Field	5: Arts and Literature; Law, Health; Technical Sciences; Humanities and Social sciences
	Level of study	6: First year; Diploma of Higher Education; BA, BS/BSc, MS/MSc, MA; Master's Degree; PhD
	Bachelor's degree	6: Mention very well with congratulations from the jury; Honours; Mention well; Mention good enough; Without honours; I do not remember
	Number of grade repetitions	8: none; 1; 2; 3; 4; 5; 6; 7
	Type of grade repetition	5: in kindergarten; in elementary school; in middle-school; in high school; at university
	Number of classes skipped	5: none; 1; 2; 3; 4
	Type of classes skipped	5: in kindergarten; in elementary school; in middle-school; in high school; at university
	Choice of field	4: Yes and I like what I study; Yes but, in the end, I don't like what I'm studying; No and I don't like what I'm studying; No, but in the end, I like what I study
**General functioning**	Initiation of social contacts	4: 0; 1 to 2; 3 to 6; more than 6
	Social isolation	3: Yes and I suffer from it; Yes but it's ok for me; No
	Hobbies	2: Yes; No
	Night out	4: Almost every evening ; Often (several evenings per week); Rarely (a few evenings per month); Almost never
	Curiosity	2: Yes; No
	Dynamism	2: Yes; No
	Daily pleasure	4: Almost all the time; Often; Rarely; Almost never
	Dwelling	7: Alone in an apartment; Alone in a dormitory; With your family; With a roommate; With a partner; In a guest room; No fixed address
	Independence	2: Yes; No
	Housework	2: Yes; No
	Grocery shopping	2: Yes; No

	Cooking	4: Yes, lunch and dinner; Yes, lunch or dinner; No, I eat at the (university) restaurant; No, someone makes me my meals
	Sedentary lifestyle	Number of hours
**Psychopathology – Validated scales**	The Beck Depression Inventory II (BDI-II)	Score
	The Temporal Experience of Pleasure Scale (TEPS)	Score
	The Rosenberg Self-Esteem Scale (RSE)	Score
**Psychopathology – Qualitative measures**	Somatic disease	2: Yes; No
	Neurodevelopmental disorders	2: Yes; No
	Psychiatric disorders	2: Yes; No
	Psychiatric disorders in relatives	2: Yes; No
	Psychotropic drugs	2: Yes; No
	Alcohol	2: Yes; No
	Frequency of alcohol intake	Number of glass per week
	Cannabis	2: Yes; No
	Frequency of alcohol intake	Number of intakes per month
	Other illicit drugs	2: Yes; No
	Fatigue	4: Always ; Often (several times per week); Rarely (once a week, or not every week); Never
	Anxiety	2: Yes; No
